# Wanderings in Search of Health

**Published:** 1890-06

**Authors:** 


*Wanderings in Search of Health.* By H. COUPLAND TAYLOR, M.D. Pp. 259. London: H. K. LEWIS. 1890.

If the natural inference from Sir Philip Sidney's remark, "All is but lip wisdom that wants experience," be permissible in respect of the work before us, we ought to derive much benefit from its perusal. The author has a most extensive personal experience of health-resorts all over the world. He writes with transparent fairness and fearlessness, handling his facts with sound judgment, and presenting them with sufficient fulness. Such a work must be of great value to those of us who are stay-at-homes; and desire, in advising our patients, to steer a safe course between the "booming" of health-resorts by interested hotel-keepers and companies, and the peevish pessimism of the literary valetudinarian. The work is most interesting to read, as well as valuable to study.

